# Rapid and simple single-chamber nucleic acid detection system prepared through nature-inspired surface engineering

**DOI:** 10.7150/thno.57153

**Published:** 2021-05-03

**Authors:** Jihyo Park, Sangwon Woo, Jiyeon Kim, Hakho Lee, Yeong-Eun Yoo, Seonki Hong

**Affiliations:** 1 Department of Emerging Materials Science, DGIST, Daegu, 42988, Republic of Korea.; 2 Department of Nano Manufacturing Technology, Korea Institute of Machinery and Materials (KIMM), Daejeon, 34103, Republic of Korea.; 3 Center for Systems Biology, Massachusetts General Hospital, Boston, MA, 02114, USA.

**Keywords:** nucleic acid-based diagnostics, injection molding, surface coating, colorimetric assay, pathogen detection

## Abstract

**Background:** Nucleic acid (NA)-based diagnostics enable a rapid response to various diseases, but current techniques often require multiple labor-intensive steps, which is a major obstacle to successful translation to a clinical setting.

**Methods:** We report on a surface-engineered single-chamber device for NA extraction and *in situ* amplification without sample transfer. Our system has two reaction sites: a NA extraction chamber whose surface is patterned with micropillars and a reaction chamber filled with reagents for *in situ* polymerase-based NA amplification. These two sites are integrated in a single microfluidic device; we applied plastic injection molding for cost-effective, mass-production of the designed device. The micropillars were chemically activated via a nature-inspired silica coating to possess a specific affinity to NA.

**Results:** As a proof-of-concept, a colorimetric pH indicator was coupled to the on-chip analysis of NA for the rapid and convenient detection of pathogens. The NA enrichment efficiency was dependent on the lysate incubation time, as diffusion controls the NA contact with the engineered surface. We could detect down to 1×10^3^ CFU by the naked eye within one hour of the total assay time.

**Conclusion:** We anticipate that the surface engineering technique for NA enrichment could be easily integrated as a part of various types of microfluidic chips for rapid and convenient nucleic acid-based diagnostics.

## Introduction

Nucleic acids (NAs) are essential biomarkers in molecular diagnostics that provide an in-depth understanding of disease-associated mechanisms and guide the corresponding treatments. For example, circulating cell-free DNA that is released from tumor cells has been suggested as a potential biomarker for cancer detection and treatment monitoring 1, 2, and identifying pathogenic DNA/RNA targets in the host allows for faster diagnoses than traditional culture-based methods 3. Among the various diagnostic tools for sequence-specific NA detection, the polymerase chain reaction (PCR) is the current gold standard with advantages in 1) signal amplification from a few copy numbers and 2) accessibility with fragmented NAs. In addition, isothermal techniques such as loop-mediated isothermal amplification (LAMP), nucleic acid sequence-based amplification (NASBA), and recombinase polymerase amplification (RPA) have emerged as alternatives to conventional thermocycling PCR in a low-resource setting 4. One limiting and underdeveloped aspect of NA-based assays is sample processing, particularly NA extraction/purification, which must be performed before NA amplification to remove inhibitors that bind to NA or interfere with DNA polymerase activity 5. Solid-phase extraction and chemical methods such as ethanol precipitation and phenol-chloroform extraction are typically used 6, but the steps are labor intensive and often manually performed.

Microfluidic devices could carry out convenient and rapid NA-based diagnostics in a sample-to-answer manner. Multiple steps, i.e., cell lysis, NA isolation/extraction and sequence-specific amplification, can be integrated into a single microfluidic chip, providing a user-friendly and portable platform 7. In addition, this platform can reduce the required sample volume down to a few microliters, which in turn decreases the assay time 7, 8. For example, H. Shao et al. developed a chip-based analysis of exosomal mRNA to monitor drug efficacy in glioblastoma multiforme 9. The chip they developed enables exosome enrichment from biofluids, lysis of and mRNA extraction from exosomes, and subsequent quantitative PCR (qPCR) in a single chip with four chambers requiring eight valves 9. W. Liu et al. designed a PCR-free NA detection chip by incorporating exothermic lysis of a virus and electrochemical detection of the viral NA; the entire process was completed within 30 min 10. T.-H. Kim et al. utilized centrifugal force for the transfer of fluid from one chamber to another in an integrated microfluidic device with five separate chambers for NA extraction, isothermal NA amplification, and subsequent read-out 11. Thermal cycler-free PCR can also be conducted in a microfluidic chip by alternately flowing the fluid across hot and cold regions 12. In addition to on-chip amplification, recently developed microfluidic devices have also been integrated with highly sensitive sensing platforms such as nano-plasmonic sensors, electrochemical sensors, and terahertz spectroscopy, enabling rapid and accurate NA identification 13-15. However, the multifunctionality built into a single chip often requires multichambers and sophisticated fluidic controls; these present a major technical obstacle to mass-producing devices, particularly via injection molding in plastics, for commercialization.

**Scheme 1:**
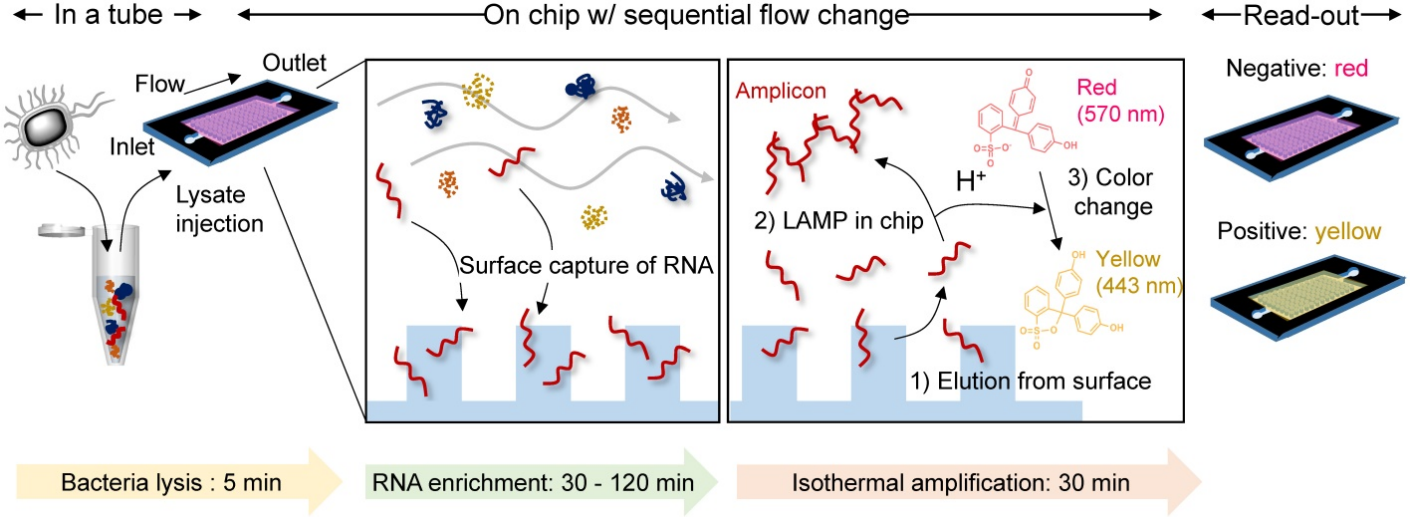
** A single-chamber system for colorimetric nucleic acid detection.** Total RNA in the bacterial lysate is captured/enriched on an engineered surface possessing a specific affinity to nucleic acids; other cellular components are removed by buffer flow. On-chip amplification of the target gene sequence is performed in the same chamber by changing the buffer to release surface-bound RNA to the chamber space filled with LAMP reagents and a pH indicator for colorimetric sensing of the target-gene amplification.

Herein, we developed a single-chamber system that executes multistep processes, including total RNA extraction, target-specific amplification, and colorimetric read-out. Silica is the most widely used substrate for solid-phase NA capture. Therefore, many efforts have been focused on fabricating silica nano-/microstructures inside the microfluidic devices. One approach has used conventional lithography techniques to chemically etch the glass or silicon substrates into pillar structures 16, 17. However, these requires multiple steps (i.e., mask fabrication, photoresist coating, UV light irradiation, chemical etching, and removal of residual photoresist) with expensive equipment and toxic chemicals, which is an obstacle to mass production in low cost. Another approach has chosen direct packing of silica beads into as-prepared microfluidic channels 9, but it is labor-intensive and hard to achieve well-ordered packing structure three-dimensionally. Different from these approaches, we chose nano-thin silica coating on as-prepared plastic devices fabricated via injection molding. Injection molding allows rapid fabrication of thermoplastics, one of the cheapest substrates used in industrial field, and therefore suitable for mass-production in low cost 18. Not only the silica but also the carboxylated surface has been reported to be effective in NA capture 19. Carboxylate groups can be simply generated on plastic surface via oxygen plasma or UV/O_3_ treatment, but also other oxygen-containing groups (i.e., -OH, -CHO, -C=O, epoxide) are generated together in uncontrollable composition and density 20. In addition, UV/O_3_ treatment has limitations in long-term storage due to its low durability 21. We reasoned that the silica coating could reduce the number of chambers required for multistep processes by providing additional reaction sites that are separated from the solution-phase reaction. The NA is captured/enriched on the engineered surface, subsequently purified by sequential exchange of the flowing buffers and is then finally released to the same chamber filled with reagents for *in situ* amplification and colorimetric read-out in a single device (**Scheme 1**). Therefore, the developed system provides a rapid and simple NA-based diagnostic platform without infectious sample transfer.

## Methods

### PC Chip fabrication

Polycarbonate plates with micropillars and chamber on the surface were fabricated by injection-molding process using a mold which a master for the micropillars and chamber were inserted inside. First, the negative structures of the micropillars and chamber were mechanically machined on a metal block by micro-milling using a micro-end mill as a durable master for large-scale production based on injection molding, which is high pressure and temperature process. The microhole, which is the negative structure of the pillar, is 200 μm in diameter and depth. The rotating speed of the micro-end mill used to machine the microhole was 40,000 rpm. Injection molding of a plate with surface microstructures consists of 3 steps generally: filling the cavity of the mold with hot melt to form the plate and to replicate surface structures, cooling the hot melt to be solidified, and releasing the molded plate. The melt temperature and mold temperature were set to 300 °C and 110 °C respectively in case of using polycarbonate as a molding material. Cycle time required to injection mold a plate was 30 seconds, which is fast enough for mass production. Using this injection mold and a thermoplastic resin such as polycarbonate, hundreds of plastic plates with a chamber and micropillars were injection-molded repeatedly and it was proved to be valid for mass production.

### Silica coating of the polycarbonate chip

The process of pDA-mediated silica coating on the chip was modified slightly from a previous study 22. Briefly, the top and bottom plates of a polycarbonate chip were incubated in 2 mg/mL dopamine hydrochloride (Sigma Aldrich) in 1× PBS (Corning®) at pH 8.3 for 3 h at room temperature with gentle shaking. After incubation, the plates were washed with distilled water and ethanol, followed by drying under nitrogen gas. Next, silica was attached to the pDA-coated plates by immersion in preactivated 100 mM tetramethyl orthosilicate (TMOS, Sigma Aldrich) in 1 mM HCl for 1 h at room temperature with gentle shaking, followed by washing with distilled water and drying under nitrogen gas.

### Surface characterization

The ad-layer and silica sequentially coated on the chip were characterized by various techniques. Scanning electron microscopy (SEM) was performed using an ultrahigh resolution field emission SEM (S-4800, Hitachi). SEM images of platinum-sputtered samples (35 s at 15 mA) were taken at high resolution to confirm the micropillar structure and morphological changes after coating. The elemental composition of the coating was analyzed using an X-ray photoelectron spectrophotometer (XPS) equipped with a monochromatic Al Kα X-ray source (1486.6 eV; anode operating at 14.8 kV and 20.5 mA) and a high resolution ESCALAB 250Xi, Thermo Scientific analyzer. The binding energies were calibrated based on the graphite C 1s peak at 284.5 eV. The vacuum pressure was approximately 10^-7^ mbar during the analysis, and the beam size was 900 µm. Water contact angle measurements were conducted using a contact angle analyzer (SEO, Korea) with a 6 μL drop of distilled water on the bare and modified surfaces. UV-visible spectra of the pDA- and silica-coated PC chip and glass were measured using a spectrophotometer (Cary 8454, Agilent) within the range from 200 to 900 nm at 2 nm intervals. To determine the coating thickness on the glass, an atomic force microscope (AFM, Park Systems Corp., XE-150) was used in noncontact mode with a high resonant frequency cantilever (force constant: 42 N/m, resonance frequency: 330 kHz, Park Systems Corp., PPP-NCHR) to scan the cross-sectional image of the scratched area of the coating with a clean cutter knife.

### Bacteria culture

*Staphylococcus aureus* (*S. aureus*) 3803 and *Escherichia coli* (*E. coli*) 1924 were obtained from the Culture Collection of Antimicrobial Resistant Microbes (CCARM), and *S. aureus* 3881 was provided by the Korean Collection for Type Cultures (KCTC). All the tested bacterial strains were cultured at 37 ℃ in tryptic soy broth (BD BactoTM) and Luria-Bertani broth (Sigma-Aldrich) for *S. aureus* and *E. coli*, respectively. The cultured bacterial pellet was collected and washed with 1× PBS prior to the on-chip bioassay.

### Total RNA extraction on the chip

Buffer sets for bacterial total RNA extraction were prepared following the methods of a previous study 28. Briefly, lysis/binding buffer was prepared by dissolving 80 mmol guanidine hydrochloride (Sigma-Aldrich) in 10 mL of nuclease-free water (Ambion®, Invitrogen) containing 0.2 mmol MES hydrate (Sigma Aldrich) and 0.2 mmol EDTA (Sigma-Aldrich) pH-adjusted to 7.0. A 3 M sodium acetate (Sigma-Aldrich) solution in nuclease-free water (pH 5.2 adjusted) and 70% v/v ethanol in nuclease-free water were used as the washing buffers. For bacterial lysis, 500 µL of lysis/binding buffer containing 1×10^8^ CFU bacteria was vortexed with 350 mg standard glass beads (diameter: 0.1 mm, Benchmark Scientific) for 5 min, and then 250 µL of ethanol was added. A 10 µL aliquot of the mixture was slowly injected into the chamber and incubated for 30 to 120 min. After removing the mixture, 450 µL of binding buffer and washing buffer were sequentially flowed through the chamber. After the residue was completely removed, the chamber was filled with nuclease-free water (total volume 10 µL) and maintained for 2 min at room temperature to elute the isolated RNA from the surface. All liquid handling with the device was performed through the simple hands-on pipetting.

### Characterization of the on-chip extracted RNA

The eluted bacterial total RNA was qualified with a 2100 Bioanalyzer (Agilent) using an RNA 6000 Pico chip and analyzed by quantitative reverse transcription PCR (RT-qPCR) using the CFX Connect Real-Time PCR system (Bio-Rad) as follows. To convert RNA to cDNA and then quantify the cDNA in a single step, a Luna® Universal One-Step RT-qPCR Kit (New England BioLabs) was used with a FAM dye-labeled probe for ribonuclease P RNA (rnpB) in *S. aureus* (Taqman® gene expression assays, ID number: Ba04646259_s1, Thermo Fisher Scientific) following the manufacturer's protocol. RT-qPCR was performed with 1 cycle of 95 °C for 10 min and 40 cycles of 95 °C for 15 s followed by 60 °C for 1 min. Quantification of gene expression was based on the cycle quantification (Cq) value compared with that of the positive controls extracted by the RNeasy Mini Kit from Qiagen. The delta Cq was calculated as (Cq of captured sample on chip) - (Cq of control sample) using CFX Maestro 1.1 software (Bio-Rad) and Microsoft Excel (Microsoft Corp., USA). The relative quantity of rnpB gene expression was determined by a delta-delta Cq calculation as 2^(-(treated sample delta Ct-control sample delta Ct)) 29.

### *In situ* colorimetric detection on the chip

Instead of eluting the surface-bound total RNA into nuclease-free water in the final step of the total RNA extraction on the chip, a mixture of WarmStart® Colorimetric LAMP Master Mix (New England BioLabs) and a customized primer set (1.6 µM for forward and backward inner primers, 0.2 µM for forward and backward outer primers, and 0.4 µM for forward and backward loop primers, Integrated DNA Technologies) was added to the chamber for on-chip amplification followed by colorimetric detection. The primer sequences for the nuc and mecA genes in MRSA are shown in **Table S2**. The chip was incubated at 63 °C for 30 min, and the color change of the solution inside the chip was monitored in real-time. During incubation, the inlet and outlet of the chip were sealed with hot glue to prevent evaporation of the solution inside, and binder clips were used to tightly secure the top and bottom plates of the chip.

### Characterization of the amplicon by gel electrophoresis

Gel electrophoresis was conducted to confirm the on-chip amplification as follows. A 2% agarose (Bioneer) solution was prepared by microwaving with 30 s pulses for 3 min. A 2 μL aliquot of 10 mg/mL ethidium bromide (EtBr) (Sigma-Aldrich) was added to 50 mL of a 2% agarose solution to visualize the DNA under UV light, and the mixture was solidified in a template. The prepared gel was placed into the gel chamber and filled with 1× TBE (Bioneer) containing 5 μL of 10 mg/mL EtBr. A 2 μL aliquot of eluted RNA from the chip was mixed with 6 μL of loading buffer (Sigma-Aldrich) and loaded carefully along with the DNA ladder (Sigma-Aldrich) into the lanes of the gel. Gel electrophoresis was conducted at 80 V for 1 h until the dye line was approximately 80% of the way down the gel. The gel was carefully placed inside a UV box (Gel documentation system, GDS-200D) to take a photographic image.

## Results and Discussion

### Thermoplastic chip fabrication by the injection-molding process

**Figure 1:**
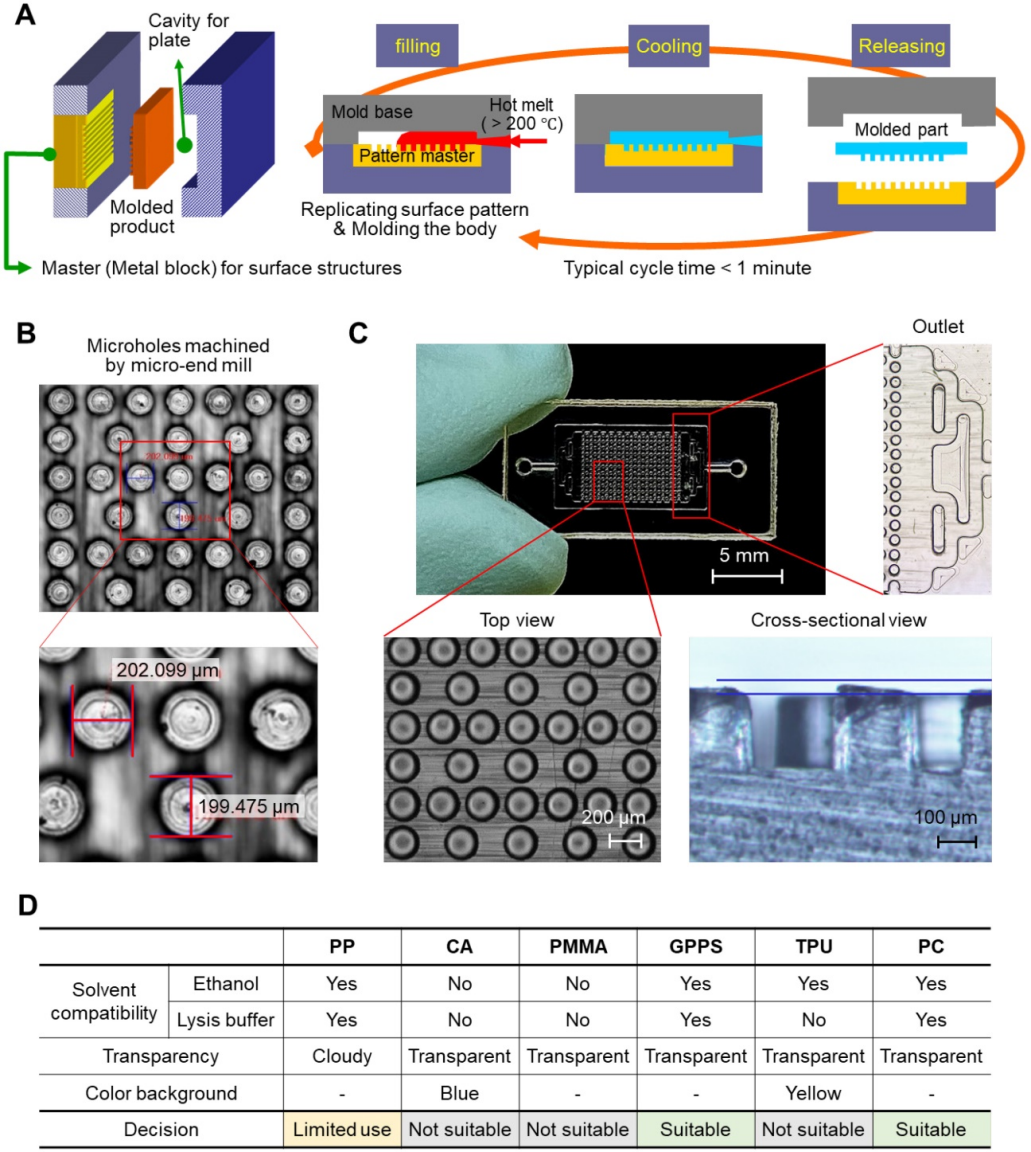
** Thermoplastic chip fabrication.** (A) A schematic illustrating the injection-molding tool and injection-molding process. (B) Microholes on the pattern master machined by a micro-end mill on a plastic mold steel block, NAK 80 (Ni-Al-Cu alloy, DAITO Steel Japan). (C) A top view and cross-sectional image of a polycarbonate chip injection molded from the pattern master with microholes. The diameter and depth of the pillars on the injection-molded chip were approximately 200 µm. (D) Thermoplastic substrate screening based on solvent compatibility, transparency, and color background.

We prepared a simple plastic chamber with micropillars to increase the surface area for NA capture. The top and bottom plates were separately fabricated and assembled after the surface modification of the inner surface of the plates with NA-attractive silica through a simple dip-coating process. The plastic plates with micropillars were injection-molded under the optimized conditions, as shown in **Figure 1A**; a hot melt of plastic (> 200 °C) was injected into the cavity of a mold, where a pattern master with negative structures of micropillars was inserted inside, and then cooled to be solidified. The entire fabrication process was completed within 30 s and scalable for the mass production of structured plastics. Microholes were machined by a micro-end mill on a steel block, NAK 80 (Ni-Al-Cu alloy, DAITO Steel Japan). The surface area of the chamber for NA capture can be increased by i) making small and tall pillars (**Figure S1**) and ii) minimizing inter-pillar distance. However, the mold machining process limits feature geometry that can be reliably cut while maintaining structural integrity: i) the lateral size ≥100 µm and ii) the aspect ratio (a, feature height / feature size) ≤1. The optimal design thus was obtained by considering the process constraints while maximizing the eventual surface area of the pillar array. The hole diameter, the negative structure of micropillar, was confirmed to be approximately 200 μm, as designed (**Figure 1B**). The depth of the microhole was also demonstrated to be approximately 200 μm based on a cross-sectional image of the micropillars that were injection-molded from the microholes (**Figure 1C, bottom**). In addition, a branched channel structure was incorporated into both the inlet and outlet for homogeneous flow control through the entire chamber space (**Figure 1C, right**).

We tested various polymeric substrates (e.g., polypropylene (PP), cellulose acetate (CA), poly(methyl methacrylate) (PMMA), general-purpose polystyrene (GPPS), thermoplastic polyurethane (TPU), and polycarbonate (PC)) for their compatibility with ethanol and cell lysis/binding buffer containing highly concentrated guanidine salt (6-8 M). As a test, we prepared a chamber with simplified channel structures only on the bottom side, and injected either ethanol or cell lysis/binding buffer into the chamber. As shown in **Figure S2**, the top plate, which was made of CA and PMMA, showed the residues that transferred from the melt channel structure on the bottom plate after incubation in both the ethanol and the cell lysis/binding buffer. The chip made of TPU melted in the cell lysis/binding buffer, although it was compatible with ethanol. PP, GPPS, and PC were confirmed as good candidates for preparing the NA extraction chip; they were compatible with both ethanol and the cell lysis/binding buffer. In addition to solvent compatibility, the transparency and color of substrates were also evaluated for colorimetric read-out (**Figure S3**). PP was a good candidate for NA extraction, but colorimetric read-out was not allowed due to its opaqueness. Finally, we chose PC as a model substrate, and GPPS can also be applicable (**Figure 1D**).

### Nature-inspired silica coating on the chamber

**Figure 2:**
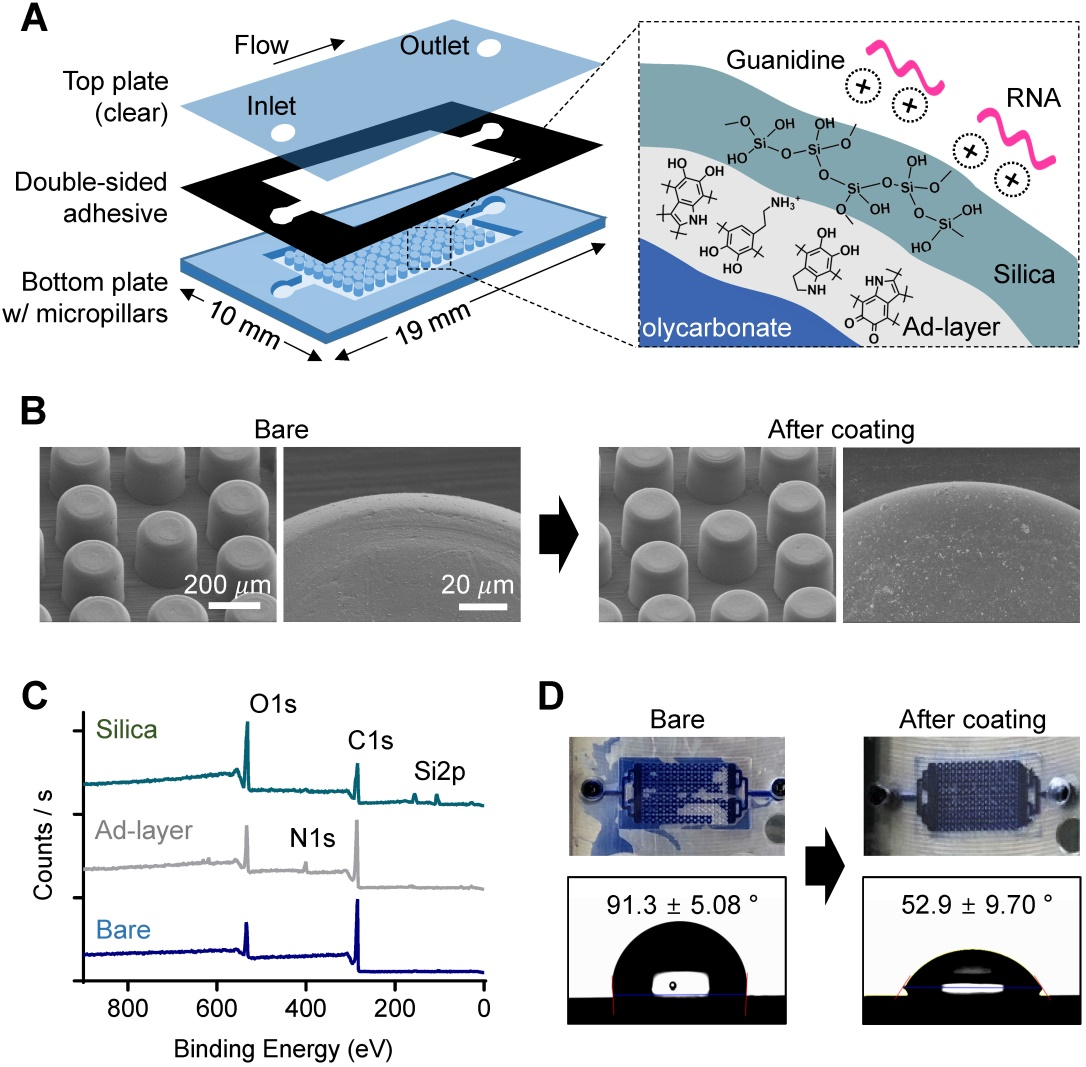
** Characterization of nature-inspired silica coating on a plastic microfluidic chip with a micropillar structure.** (A) A schematic illustrating the single-chamber chip made of polycarbonate (PC). The bottom plate structured with micropillars (diameter: 200 µm, height: 200 µm) was modified with nucleic acid-enrichable silica by utilizing pDA as a universal adhesive layer (ad-layer). (B) Surface roughness change in micropillars after silica coating, as analyzed by scanning electron microscopy (SEM). There was no visible difference at the microscale, but some aggregates of silica were attached to the surface in the enlarged view after coating. (C) Elemental analysis of the top surface confirming ad-layer deposition and silica coating, as analyzed by X-ray photoelectron spectroscopy (XPS). (D) Enhancement of hydrophilicity on the surface of the chip after silica coating, as confirmed by a water flow check (top, applied volume: 10 µL) and static water contact angle measurement (bottom).

Thermoplastics have poor chemical and biological availability due to their intrinsic hydrophobicity and lack of nucleophilic functional groups for bioconjugation. To prepare our device for NA assays, we modified the device surface through a bioinspired silica coating process 22. Silica is necessary in NA-based bioassays due to its intrinsic affinity to NA in the presence of chaotropic agents, but silica's hydrophilicity prevents its direct attachment/coating to hydrophobic thermoplastics. To solve this problem, we added a mussel-inspired polydopamine (pDA) coating as an intermediate adhesive layer that linked hydrophobic and chemically inert polycarbonate to the hydrophilic silica (**Figure 2A**, right). The coating process was simple and environmentally friendly; each coating was performed by simple dipping in an aqueous solution containing dopamine for 3 h and then washing, followed by immersion in an aqueous solution containing prepolymerized tetramethyl orthosilicate (TMOS) for another 1 h, washing, and drying, as reported in our previous study 22. Simple dipping process has advantages in mass production as multiple samples can be coated at once in a batch. We coated the bottom plate of the chamber with micropillars separately and then assembled it with the top plate via a commercial double-sided adhesive because PC is rarely permeable to oxygen, while the pDA coating is accelerated by an oxygen supply (i.e., contact with the air) 23 (**Figure 2A**, left). We compared the RNA capture efficiency of cases 1) when both the top and bottom plates were coated, and 2) when only the bottom plate with the micropillars was coated (**Figure S4**). The difference was insignificant (*P* = 0.1855, unpaired *t*-test), but the both-side coated chip showed slightly less captured RNA than the bottom-side coated chip. A plausible explanation is that the increased hydrophilicity of the top plate may increase the flow through the gap between the top plate and the top of the micropillars rather than through the side area of the micropillars on the bottom plate.

The change in surface morphology after pDA and silica coating was analyzed using scanning electron microscopy (SEM). As shown in **Figure 2B**, the microstructures did not change, but submicron silica aggregates were on the pillar surface after the silica coating, which can promote NA capture efficiency by increasing the surface area, as previously confirmed 22. The chemical elemental change on the surface of the bottom plate after pDA coating and subsequent silica deposition was then analyzed using X-ray photoelectron spectroscopy (XPS) (**Figure 2C**); a nitrogen 1s peak was detected after pDA coating, and silica 2p peaks were observed with an increased oxygen 1s peak to carbon 1s peak ratio after silica (SiO_2_) deposition. In addition, high-resolution C1s spectra showed dramatic changes in functional group composition of the chip surface; the peak at 289.0 eV corresponding to the aromatic groups of bare PC was almost disappeared after pDA and silica deposition, indicating the pDA/silica coating can be thicker than few tens nanometer, the detectable depth limit of XPS (**Figure S5**). Finally, surface engineering resulted in a dramatic decrease in the hydrophobicity of the PC surface (**Figure 2D**); the silica-coated chamber was successfully filled with aqueous fluid (right), but air bubbles were randomly trapped in the bare PC chamber (left). The static contact angle measured on a plain substrate without the micropillar structure also decreased from 91.3 ± 5.08° to 52.9 ± 9.70° after silica coating (bottom).

**Figure 3:**
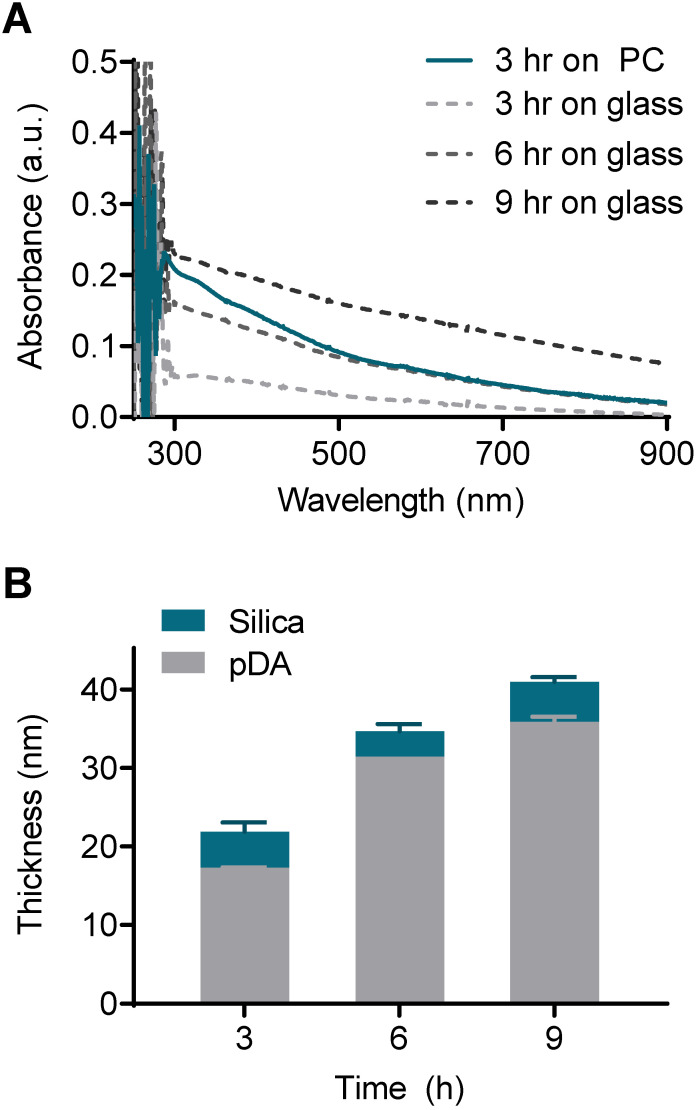
** Evaluation of coating thickness on the PC chip.** (A) UV-vis absorbance spectra of the PC chips and glass slides coated with pDA as an ad-layer for 3, 6, and 9 h. (B) Thickness of the ad-layer and sequential silica coating on glass slides as measured by atomic force microscopy (AFM).

The pDA and silica coating thickness was on the scale of tens of nanometers, and this scale required measurement using atomic force microscopy (AFM). However, the direct AFM measurement was incompatible with thermoplastics due to their intrinsic rough nature. As an alternative approach, we deposited silica on the flat glass and measured the coating thickness with AFM. Then, the thickness of the pDA coating on the PC could be indirectly determined by comparing the UV-vis spectrum with that on the glass with a known thickness. The absorbance of the pDA on the PC coated for 3 h was comparable to that of the glass coated for 6 h across a wide range of wavelengths (from 500 nm to 900 nm) (**Figure 3A**). A difference in absorbance below 500 nm was observed due to the intrinsic difference in the absorbance of the bare substrates (**Figure S6**). The pDA thickness increased from 17 nm to 36 nm as the coating time increased from 3 h to 9 h on the glass substrate, and the thickness of the silica deposited on the pDA was approximately 4 nm regardless of the pDA thickness (**Figure 3B**). Overall, the thickness of the entire coating on the PC substrate was assumed to be approximately 35 nm.

### Bacterial total RNA capture

We next evaluated the NA capture efficiency of the silica-coated chamber. As a lysis/binding buffer, we used a combination of highly concentrated guanidine salt (GuHCl, 8 M) and ethylenediaminetetraacetic acid (EDTA, 20 mM) (**Figure S7**). Guanidine is a well-known chaotropic agent that promotes NA binding to a silica surface 24. EDTA chelates divalent ions such as Mg^2+^, Ca^2+^, and Mn^2+^, which are required for enzymatic activity of RNase, and thereby prevents RNA degradation by RNase 25, 26. As shown in **Figure S7**, the RNA capture efficiency on the chip was at its maximum when both GuHCl and EDTA were used (10^6^ CFU bacteria tested) compared to the single component lysis/binding buffer (GuHCl buffer: 53%, EDTA buffer: 35% to GuHCl/EDTA buffer). **Table S1** shows a comparison of the RNA extraction steps using a commercial silica filter system (RNeasy Mini Kit, Qiagen) and our silica-coated microfluidic chip. The cell lysis process was similar, but the washing steps with centrifugal filtration in the commercial kit were replaced by the simple injection of buffers. Finally, the elution volume was reduced from 30-50 μL to 10 μL, which provides a higher concentration of eluted total RNA.

**Figure 4:**
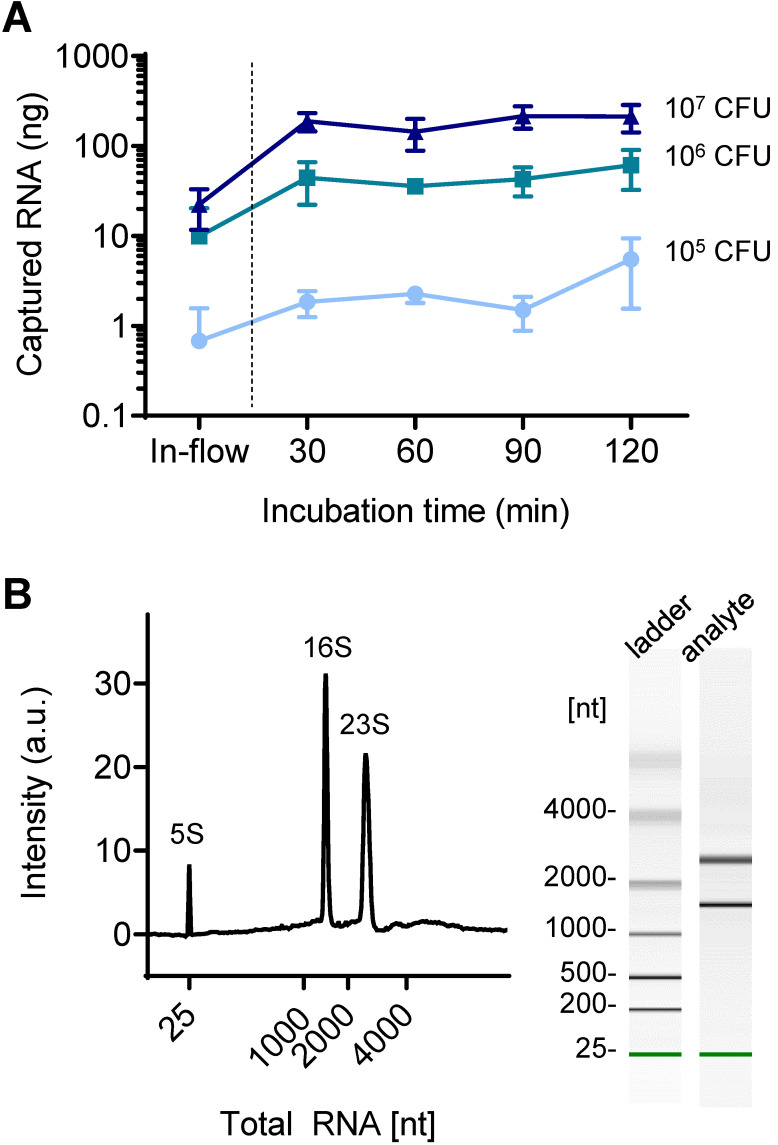
** Total RNA capture in a silica-modified microfluidic chamber.** (A) Quantitative analysis of surface-captured total RNA for different applied bacteria counts as the incubation time increases. (B) Quality of surface-captured total RNA as analyzed by a bioanalyzer (total RNA concentration: 784 pg/µL).

While conventional centrifugal filter devices are performed in-flow, the fabricated device in this study required static incubation for at least 30 min to achieve sufficient capture efficiency. As shown in **Figure 4A**, the 30 min-incubation dramatically increased the captured amount of NA compared to in-flow capture: 0.7 ng to 1.8 ng in 10^5^ CFU of bacteria tested (270% increase), 9.9 ng to 44 ng (447% increase) in 10^6^ CFU, and 22 ng to 189 ng in 10^7^ CFU (845% increase). A further increase in incubation time up to 120 min also slightly increased the on-chip captured RNA when using a small amount of RNA (10^5^ CFU: 1.8 ng to 5.5 ng, 10^6^ CFU: 44 ng to 61 ng), but the increase was minimal at high concentrations of RNA (10^7^ CFU: 189 ng to 214 ng). The captured NA was quantified by reverse transcription quantitative PCR (RT-qPCR) after elution into 10 μL of nuclease-free water. Surface capture of analyte in flow is mediated by Brownian motion, and the increased incubation time contributes to increase the chance of NA moving toward the pillar surface, which is in the line with our previous report 27. On the other hand, upper limit of the NA capture capacity is determined by the surface area. The total RNA capture capacity of the nature-inspired silica coating we developed was previously reported to be 0.357 μg/cm^2^ 22. Based on this, it is assumed that more than 200 ng of total RNA can be captured in the current device setting. While the sensitivity improved as the capture efficiency increased, the longer incubation time and thereby the longer total assay time (i.e., NA extraction and on-chip amplification followed by read-out) can be an obstacle to rapid diagnosis in a point-of-care setting. Many of recently reported integrated devices for sample-to-answer NA-based diagnosis required a total reaction tome of 30 to 100 min, even though the microfluidics and/or centrifugal disc platforms were chosen (**Table S2**). Therefore, this incubation time can still be acceptable for rapid diagnostics.

The quality of on-chip extracted bacterial total RNA was further assessed using an automated gel electrophoresis system (Bioanalyzer, Agilent) (**Figure 4B**). The on-chip purified total RNA retained its structural integrity and quality; the characteristic bacterial ribosomal RNAs (5S, 16S, and 23S) were detected. It was confirmed that the coating was bioinert; there was no leaching of chemicals that degrade NAs.

### On-chip colorimetric LAMP assay

**Figure 5:**
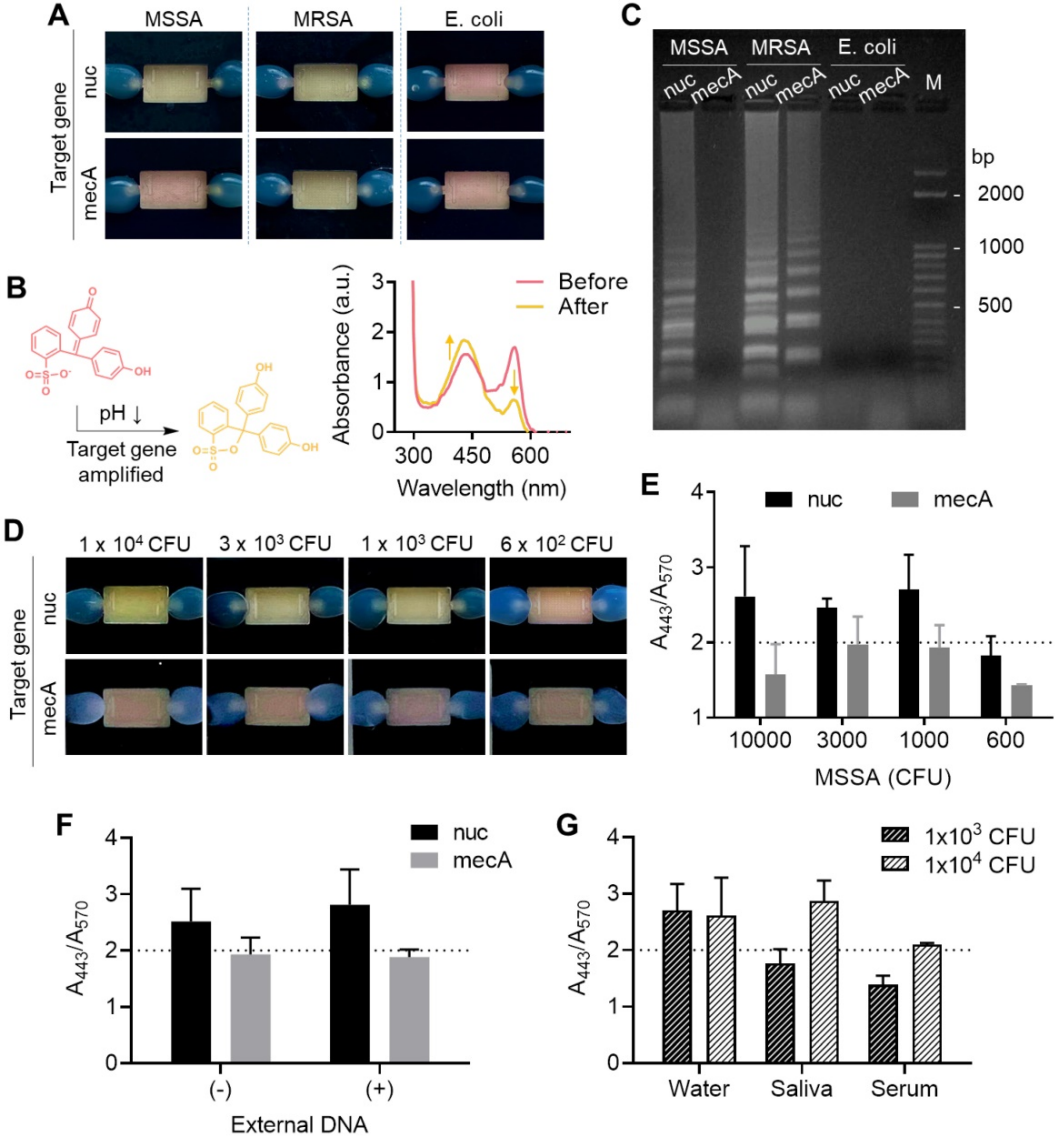
** Colorimetric detection of on-chip enriched RNA amplification in the chamber.** (A) Pink-to-yellow color change of the pH indicator (phenol red) as target gene amplified after on-chip bacterial total RNA enrichment (10^5^ CFU was used in each chamber). (B) Visible range absorbance spectra change after target gene amplification. (C) Agarose gel electrophoresis of on-chip amplified target genes of MSSA, MRSA, and *E. coli*. (D) Detection limit of the on-chip colorimetric detection system by the naked eye. (E) Quantitative analysis of the colorimetric signal by measuring the ratio of absorbance at 443 nm and absorbance at 570 nm. (F) Quantitative colorimetric detection of nuc and mecA genes in 1×10^3^ CFU MSSA in the presence and absence of external DNA in bacterial lysate (5 µg/mL, herring testes DNA). (G) Comparison of detection limit in bacteria spiked in biofluids as mock clinical samples.

Finally, *in situ* target gene amplification was performed following on-chip RNA enrichment without any sample transfer. To do so, an aqueous solution containing a polymerase (WarmStart® Colorimetric LAMP Master Mix, New England BioLabs) and a customized primer set (**Table S3**) were directly injected into the chamber with surface-bound RNA as the elution buffer, and the chip filled with the buffer was directly incubated in a conventional heater at 63 °C for 30 min instead of collecting the buffer out. We coupled a colorimetric pH indicator, phenol red, for naked-eye detection of target gene amplification, which generates protons inducing a color change of the pH indicator from red to yellow. As a demonstration, the nuc and mecA genes were separately amplified on the chip after bacterial total RNA was enriched. The nuc gene distinguishes gram-positive *Staphylococcus aureus* (*S. aureus*) from gram-negative *Escherichia coli* (*E. coli*), and mecA gene indicates resistance to the antibiotic methicillin of *S. aureus*. As shown in** Figure 5A**, the color changes well-matched with the presence of target genes for each bacterial strain (methicillin-resistant *S. aureus* (MRSA): nuc^+^ and mecA^+^, methicillin-sensitive *S. aureus* (MSSA): nuc^+^ and mecA^-^, *E. coli*: nuc^-^ and mecA^-^) (10^5^ CFU used). The color change was also observed by measuring absorbance at visible range; the absorbance at 443 nm increased while the absorbance at 570 nm decreased (**Figure 5B**). Finally, the successful on-chip amplification of bacterial RNA was confirmed by conventional gel electrophoresis (**Figure 5C**). The total RNA capture efficiency was retained for the second trial of the used chip (98% to 1st trial) but finally decreased to 68.4% at third use (**Figure S8**). Therefore, the chip is suitable for a single use.

We next measured the limit of detection of the colorimetric bacterial gene by a surface-engineered single-chamber system. As shown in **Figure 5D**, up to 1×10^3^ CFU of MSSA was successfully detected within an hour of the total assay time (i.e., 30 min RNA extraction and 30 min LAMP) by the naked eye without any additional equipment required. The color change was also quantitatively analyzed by measuring the ratio of absorbance at 443 nm and 570 nm (**Figure 5E,** UV-vis absorbance spectra is shown in**Figure S9**). Silica has affinity to both RNA and DNA. Therefore, we performed another experiment to see whether the competitive binding of DNA and RNA affects to the sensitivity of on-chip colorimetric assays. As shown in **Figure 5F** and**Figure S10**, the limit of detection, i.e., the lowest bacteria number detectable through the on-chip colorimetric assays (1×10^3^ CFU), did not altered in the presence of external DNA added to the bacterial lysate (5 μg/mL, herring testes DNA from Sigma Aldrich). It may be due to the large surface area that sufficiently captures detectable amount of RNA even in the presence of external DNA. In fact, our total RNA capture procedure includes a washing step with sodium acetate (3 M, pH 5.2), which removes DNA from the surface and selectively remains RNA bound to the surface 28. Therefore, it was confirmed that the surface bound NA once after the washing step was mostly RNA not DNA, which was non-degradable to DNase I treatment (**Figure S11**). Finally, we tested the limit of detection of bacteria spiked in human serum and saliva as mock clinical samples (**Figure 5G** and**Figure S12**). The sensitivity was lower in these samples compared to bacteria spiked in pure water; it was failed to detect 1×10^3^ CFU in both saliva and human serum, but 1×10^4^ CFU was detectable in saliva. The case of 1×10^4^ CFU in human serum showed orange color, indicating insufficient pH change from red to yellow, but still the color was distinguishable from the yellow negative controls. The performance of our device (i.e., limit of detection, total assay time) was comparable with recently developed integrated devices for nucleic acid-based pathogen detection in sample-to-answer manner (**Table S4**). Other types of amplification systems such as recombinase polymerase amplification (RPA), isothermal helicase dependent amplification (tHDA), and strand exchange amplification (SEA) with various read-out platform in fluorescence and lateral flow system can also be easily integrated with our system without changes in device setting.

## Conclusion

We have implemented a plastic microfluidic device for on-chip NA extraction and amplification. The device was fabricated through thermoplastic injection-molding, enabling scaled-up production. For efficient NA capture, we embedded micropillars and coated their surfaces with nanometer-thick silica. The engineered surface indeed showed a high affinity to NA in the presence of a chemotropic agent while remaining chemically and biologically inert during the entire process, i.e., NA extraction and polymerase-based isothermal amplification at 63 ℃. We expect that the developed module can easily be integrated into various types of multifunctional microfluidic devices for rapid and convenient diagnostics.

## Supplementary Material

Supplementary figures and tables.
