# Supplementary material for: Rapid and simple single-chamber nucleic acid detection system prepared through nature-inspired surface engineering
Source: Theranostics. 2021 May 3;11(14):6735–45. doi: 10.7150/thno.57153 (PMC8171086; doi:10.7150/thno.57153)
Supplement: Supplementary file 1 — Supplementary figures and tables. [file thnov11p6735s1.pdf]

Rapid and simple single-chamber nucleic acid detection system prepared through nature-inspired surface engineering

Jihyo Park[1], Sangwon Woo[2], Jiyeon Kim[1], Hakho Lee[3]*, Yeong-Eun Yoo[2]*, Seonki Hong[1]*

[1]Department of Emerging Materials Science, DGIST, Daegu, 42988, Republic of Korea

[2]Department of Nano Manufacturing Technology, Korea Institute of Machinery and Materials (KIMM), Daejeon, 34103, Republic of Korea

[3]Center for Systems Biology, Massachusetts General Hospital, Boston, MA, 02114, USA

*Correspondence to: hlee@mgh.harvard.edu (H.L.), yeyoo@kimm.re.kr (Y.-E.Y.), seonkihong@dgist.ac.kr (S.H.)

Supplementary Materials

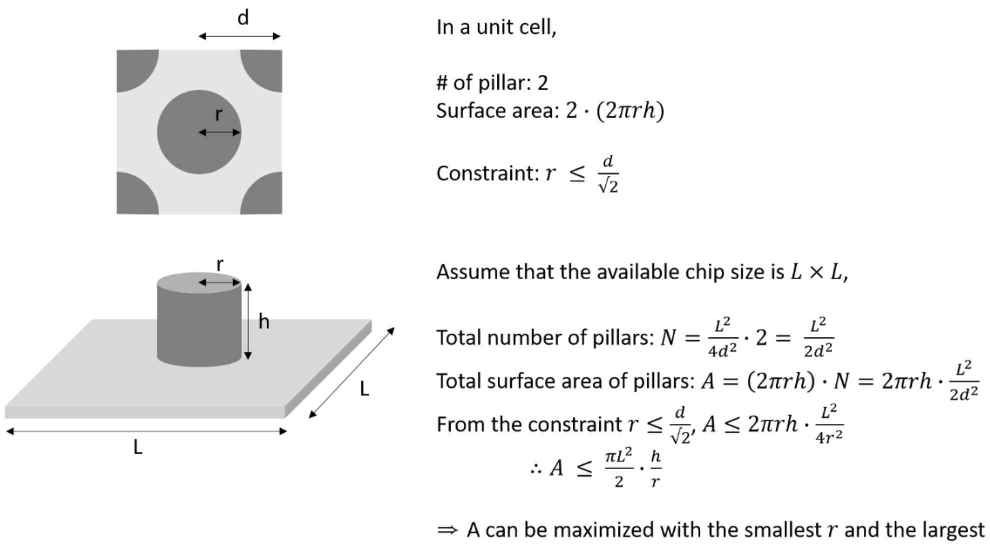

Figure S1. Calculation of the surface area of pillar array indicating its dependency on the pillar geometry.

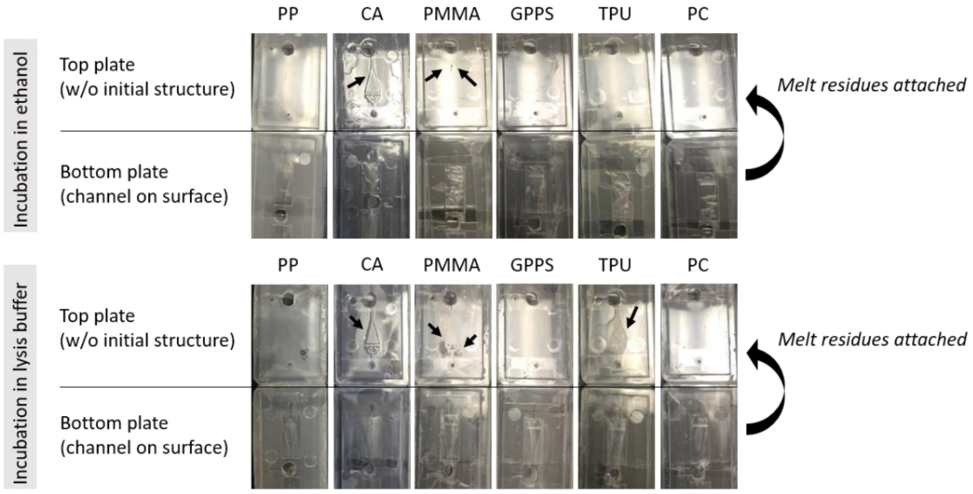

Figure S2. Solvent compatibility test of plastic chips composed of various substrates. Melt residues of the bottom plate composed of CA, PMMA, and TPU incubated in ethanol or cell lysis buffer were attached to the top plate, which had no initial structure.

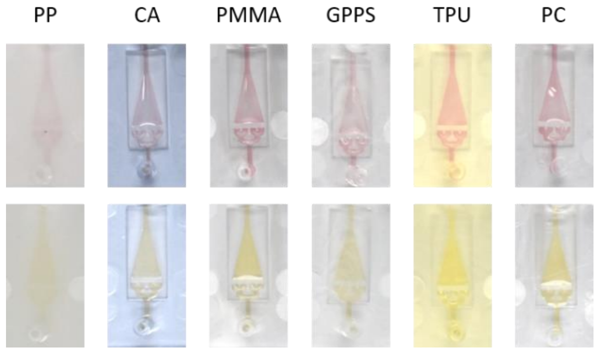

Figure S3. Transparency and color background of plastic chips composed of various substrates.

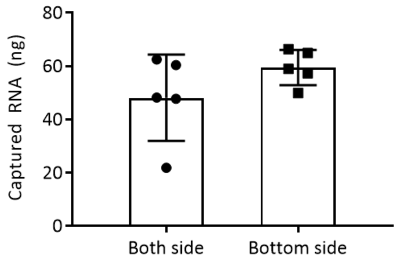

Figure S4. Total RNA capture efficiency comparison of the bottom-side coating and both-side coating on the chip. Micropillars were only fabricated on the bottom side.

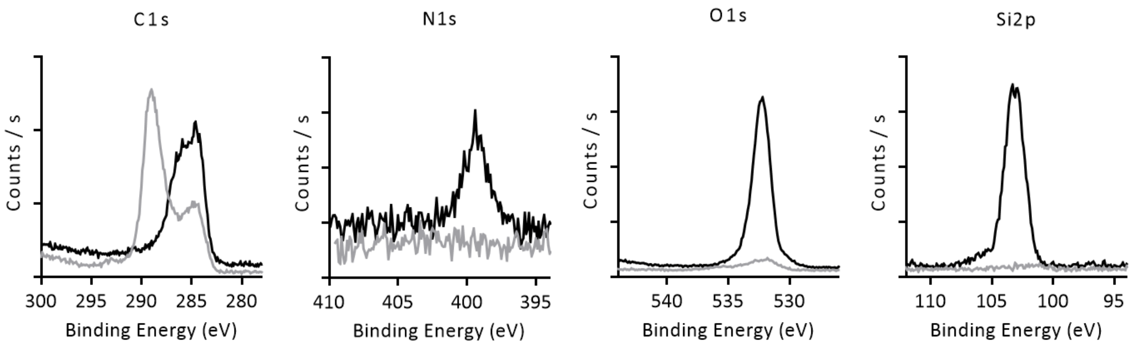

Figure S5. High-resolution XPS spectra of elements before and after pDA/silica coating (grey: bare PC substrate, black: pDA/silica coated PC).

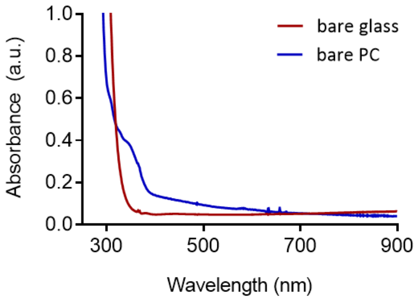

Figure S6. UV-vis spectrum of the bare PC chip and the bare glass.

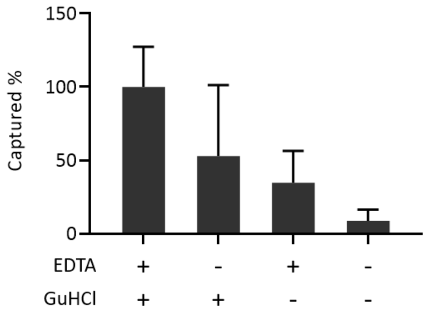

Figure S7. The optimization of the cell lysis buffer composition (10^6 CFU, 30 min incubation).

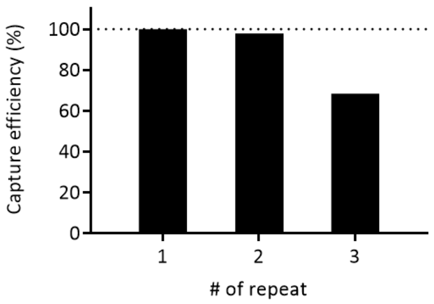

Figure S8. Total RNA capture efficiency after repeated use of the chip.

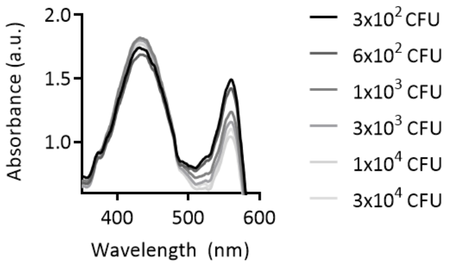

Figure S9. UV-vis absorbance spectra of on-chip analyzed samples with colorimetric dye.

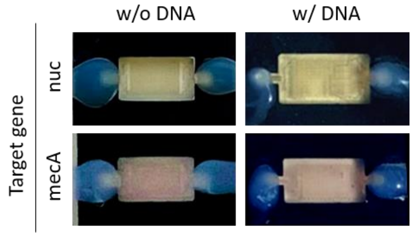

Figure S10. Colorimetric detection of nuc and mecA genes in 1×10^3 CFU MSSA in the presence and absence of external DNA in bacterial lysate (5 µg/mL, herring testes DNA).

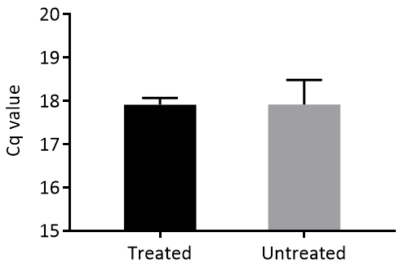

Figure S11. DNase I treatment on surface-bound NA after the washing step.

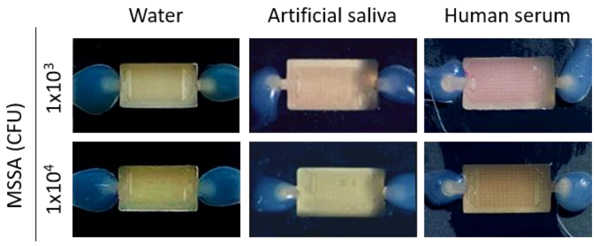

Figure S12. Comparison of detection limit in bacteria spiked in biofluids as mock clinical samples.

	RNeasy mini kit & column device		On-chip RNA extraction	
**Lysis**	Treat bacterial sample with RNAprotect Bacteria Reagent	In microtube	Disperse up to 10^8 cells in 500 μL of lysis buffer	In microtube
	Disperse up to 10^8 cells in 350 μL of buffer RLT			
	Transfer the lysate to the device			
	5 min vortexing with glass beads		5 min vortexing with glass beads	
	Add an equal volume of 70% ethanol to the lysate		Add 250 μL of 98% ethanol to the lysate	
**RNA binding**	Transfer up to 700 μL to the device	In centrifugal filter column	Inject 10 μL to the chip	In single-chamber chip
	Centrifuge 15 sec at 8,000 x g		Incubate for 30-120 min at static, rt	
**Washing**	Add 700 μL of buffer RW1		Inject 450 μL of sodium acetate buffer	
	Centrifuge 15 sec at 8,000 x g			
	Add 500 μL of buffer RPE		Inject 320 μL of 70% ethanol	
	Centrifuge 15 sec at 8,000 x g			
	Add 500 μL of buffer RPE			
**Dry**	Centrifuge 2 min at 8,000 x g		Withdraw to remove residual solvents	
**Elution**	Add 50 μL of RNase-free water		Inject 10 μL of RNase-free water	
	Incubate for 1 min at static, rt		Incubate for 2 min at static, rt	
	Centrifuge 1 min at 8,000 x g		Collect the eluate	

Table S1. Comparison of on-chip RNA extraction developed in this study and a commercial total RNA extraction kit from Qiagen (RNeasy mini).

References	Device fabrication					Sample type	Limit of detection	Total reaction time
	Substrate	Fabrication method	Number of chambers	Device format	NA purification			
This study	PC	Injection molding	Single	Microfluidic chip	Nano-thin silica coating	Lysate	10^3-10^4 CFU	60 min
Lab Chip, 19, 3804–3814 (2019)	PDMS	Soft lithography	Multiple	Microfluidic chip	-	Whole bacteria	30 CFU	40 min
Lab Chip, 18, 610-619 (2018)	PMMA	CNC milling	Multiple	Centrifugal disc	Zeolite beads*	Lysate	960 CFU	70 min
Lab Chip, 20, 384-393 (2020)	PC	Injection molding	Multiple	Microfluidic chip	Silica-coated magnetic beads	Whole bacteria	2×10^2-2×10^3 CFU	100 min
Sci Rep, 7, 1460 (2017)	PMMA	CNC milling	Multiple	Centrifugal disc	-	Whole bacteria	10^2-10^3 CFU	70 min
Biosens Bioelectron, 91, 334–340 (2017)	PC/PMMA	CNC milling	Multiple	Centrifugal disc	Silica beads	Lysate	50 CFU	80 min
Anal Chem, 86, 3841–3848 (2014)	Polycarbonate	CNC milling	Multiple	Centrifugal disc	-	Whole bacteria	10^1-10^2 CFU	30 min
RSC Adv, 4, 42245-42251 (2014)	Paper or porous polymer monoliths	-	Single	Pipette tip	Alcohol precipitation	Whole bacteria	10^3 cells	50 min
Anal Bioanal Chem. 412, 6927-6923 (2020)	Silica	-	Single	Membrane filter	Silica membrane	Lysate	-	50 min

Table S2. Comparison with recently developed integrated devices for nucleic acid-based bacteria detection in sample-to-answer manner. *used to adsorb impurities not NA.

	nuc gene	mecA gene
F3	5’-TCGCTTGCTATGATTGTGG-3’	5’-GGTACAAGATGATACCTTCGTT-3'
B3	5’-ACATACGCCAATGTTCTACC-3'	5’-ATAGCAGTACCTGAGCCAT-3'
FIP	5’-GTACAGTTTCATGATTCGTCCCGCCATCATTATTGTAGGTGT-3'	5’ –TCTTCAGAGTTAATGGGACCAAACAGAAAGTCGTAACTATCCTC-3'
BIP	5’-TGTTCAAAGAGTTGTGGATGGTGTACAGGCGTATTCGGTT-3'	5’-AAGCTCCAACATGAAGATGGCTTGTATGTGCGATTGTATTGC-3'
FLP	5’-TTGAAAGGACCCGTATGATTCA-3'	5’-ACCTAATAGATGTGAAGTCGCT-3'
BLP	5’-GATACGCCAGAAACGGTGA-3'	5’-CGTGTCACAATCGTTGACG-3'

Table S3. LAMP primer set for nuc and mecA genes in *S. aureus* [1].

References	Sample preparation		Limit of detection			Amplification	Read-out	Total reaction time
	Sample	Loading type	Bacteria	Per reaction	Concentration			
This study	Water or saliva	Lysate	*S. aureus*	10^3-10^4 CFU	10^2-10^3 CFU/uL	LAMP	Colorimetric	60 min
Lab Chip, 19, 3804–3814 (2019)	-	Whole bacteria	*S. aureus*	30 CFU	1.5 CFU/uL	LAMP	Colorimetric	40 min
Lab Chip, 18, 610-619 (2018)	Spiked in urine	Lysate	*P. aeruginosa*, *S. typhimurium*	960 CFU	$2x10^2$ cells/uL	LAMP	Fluorescence	70 min
Lab Chip, 20, 384-393 (2020)	Spiked in urine	Whole bacteria	*E. coli*, *S. aureus*	$2x10^2$-$2x10^3$ CFU	1-10 CFU/uL	LAMP	Fluorescence	100 min
Sci Rep, 7, 1460 (2017)	Spiked in human serum	Whole bacteria	*S. aureus*, *S. typhimurium*	10^2-10^3 CFU	1-10 CFU/uL	LAMP	Fluorescence	70 min
Biosens Bioelectron, 91, 334–340 (2017)	Tap water or milk	Lysate	*S. typhimurium*	50 CFU	10 CFU/uL	LAMP	LFA	80 min
Anal Chem, 86, 3841–3848 (2014)	Spiked in PBS or milk	Whole bacteria	*S. enteritidis*	10^1-10^2 CFU	10^1-10^2 CFU/mL	RPA	LFA	30 min
RSC Adv, 4, 42245-42251 (2014)	Spiked in urine	Whole bacteria	*C. trachomatis*	10^3 cells	10 cells/uL	tHDA	LFA	50 min
Anal Bioanal Chem, 412, 6927-6923 (2020)	Spiked in oyster	Lysate	*V. parahaemolyticus*	-	10^3 CFU/g	SEA	Fluorescence	50 min

Table S4. Comparison with recently developed integrated devices for nucleic acid-based bacteria detection in sample-to-answer manner**.** (LAMP, loop-mediated isothermal amplification; RPA, recombinase polymerase amplification; tHDA, isothermal helicase dependent amplification; SEA, strand exchange amplification; *S. aureus, staphylococcus aureus; E. coli, Escherichia coli; P. aeruginosa, Pseudomonas aeruginosa; S. typhimurium, Salmonella typhimurium; S. enteritidis, Salmonella enteritidis; C. trachomatis, Chlamydia trachomatis; V. parahaemolyticus, Vibrio parahaemolyticus)*

Reference

[1] Hong S, Park KS, Weissleder R, Castro CM, Lee H. Facile silicification of plastic surface for bioassays. Chem Commun. 2017; 53(13): 2134-7.
